# An unusual cause of significantly elevated blood alpha-fetoprotein levels: a case report and literature review

**DOI:** 10.3389/fonc.2024.1393074

**Published:** 2024-05-15

**Authors:** Yue Zheng, Jianping Yao, Jin Liu, Feimin Zhao

**Affiliations:** ^1^ Department of General Medicine, The Fifth School of Clinical Medicine of Zhejiang Chinese Medical University, Huzhou Central Hospital, The Affiliated Central Hospital of Huzhou University, Huzhou, Zhejiang, China; ^2^ Department of Endocrinology, The Fifth School of Clinical Medicine of Zhejiang Chinese Medical University, Huzhou Central Hospital, The Affiliated Central Hospital of Huzhou University, Huzhou, Zhejiang, China; ^3^ Department of Pathology, The Fifth School of Clinical Medicine of Zhejiang Chinese Medical University, Huzhou Central Hospital, The Affiliated Central Hospital of Huzhou University, Huzhou, Zhejiang, China

**Keywords:** alpha-fetoprotein, gastric cancer, gastric hepatoid adenocarcinoma, metastasis, clinical application

## Abstract

Alpha-fetoprotein (AFP) serves as a crucial diagnostic marker for primary hepatocellular carcinoma (HCC) and germ cell tumors (GCTs), with rare instances of significantly elevated levels in other diseases. In this study, we present a case of an elderly patient who was diagnosed with AFP-producing gastric cancer (AFPGC) following an elevated AFP result during physical examination. In investigating liver cancer at an early stage, the diagnosis was missed because of failure in detecting the lesion, resulting in delayed treatment initiation. AFPGC is a rare aggressive tumor that demands heightened awareness among clinicians to foster early detection, diagnosis, and treatment for improved prognosis.

## Introduction

Alpha-fetoprotein (AFP) is a glycoprotein belonging to the albumin family, which is synthesized by fetal hepatocytes and yolk sacs. It serves as a major diagnostic marker for HCC and GCTs, exhibiting slight elevation in hepatitis and cirrhosis, and occasional elevation in other tumors such as gastric cancer and bowel cancer (1). This paper presents a case of a healthy elderly male who incidentally showed a significant increase in blood AFP levels during physical examination. Early detection focused on liver cancer screening. However, no lesion was detected and the diagnosis was missed. Three months later, the patient was hospitalized because of “anemia”, and AFPGC was discovered. AFPGC is a rare gastric cancer with an incidence rate of 1.3%-5.4% (2), and with median survival rate of 10.3 months (3). The 5-year survival rate of patients with advanced AFPGC is only 4.4% (4), indicating a very poor prognosis.

## Case presentation

**Figure 1:**
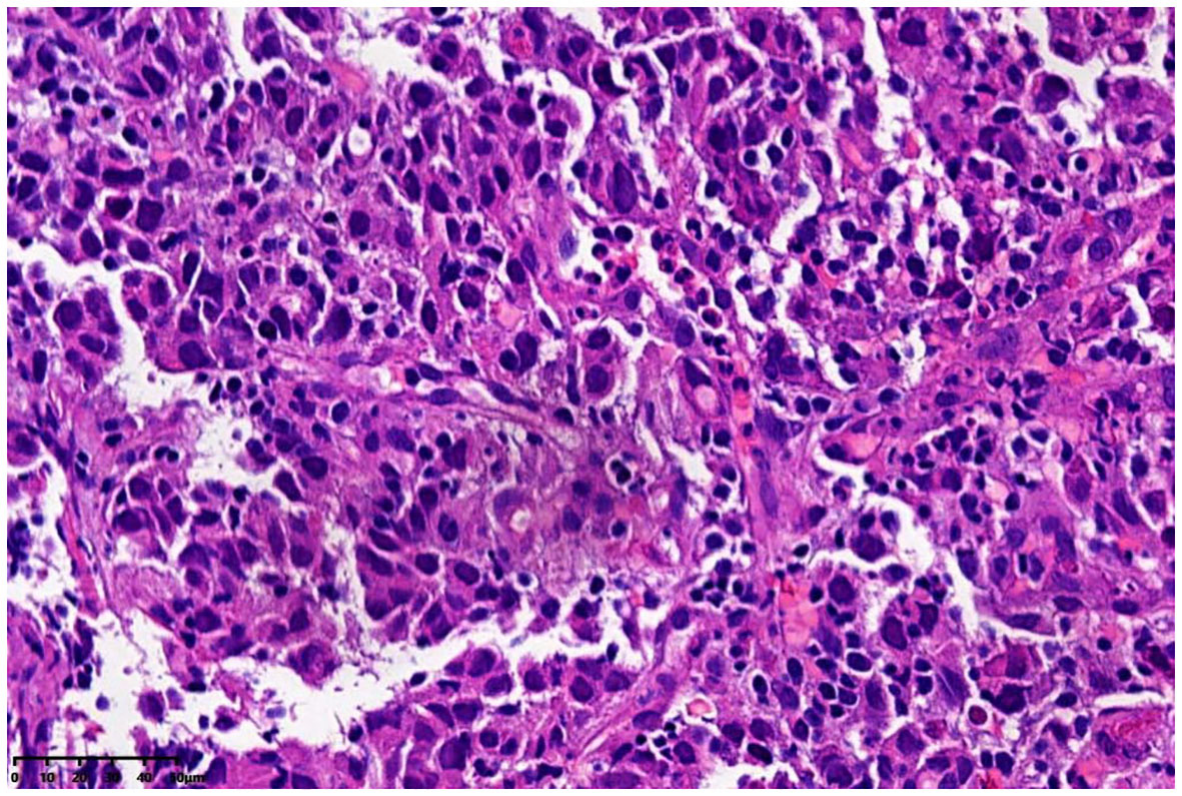
Gastroscopy pathology.

**Figure 2:**
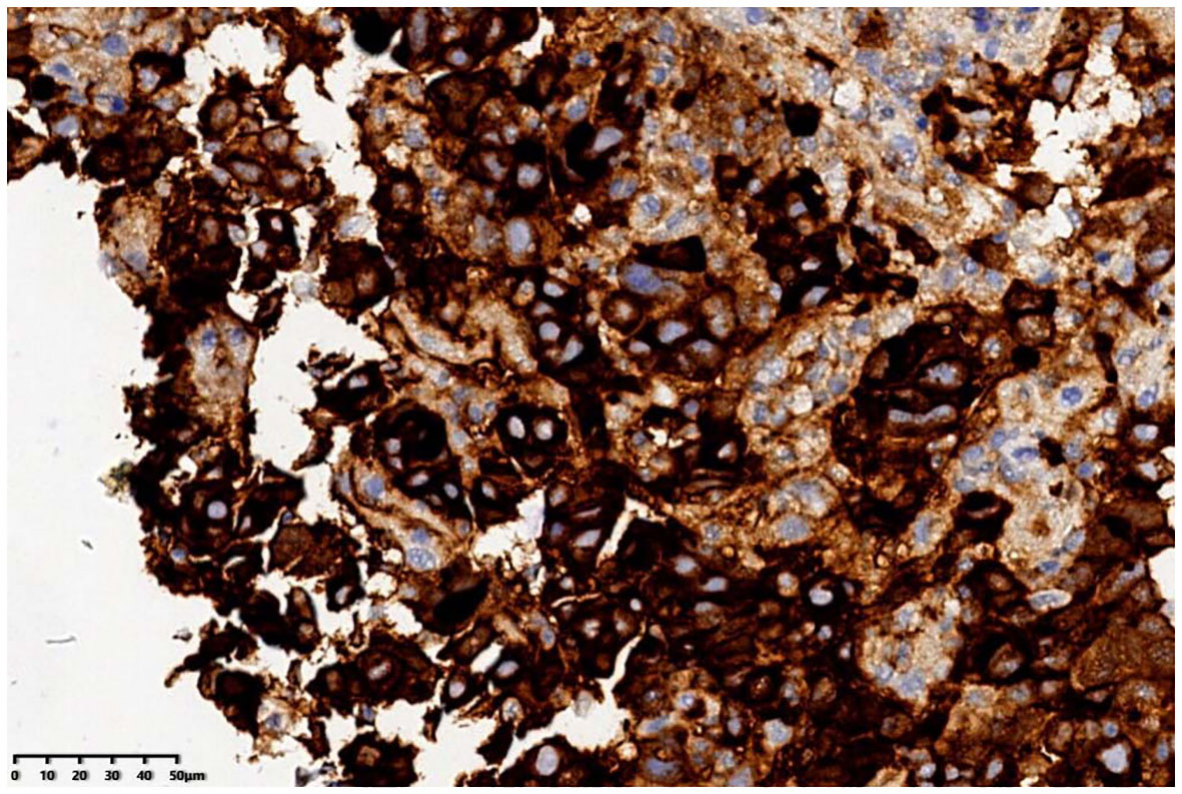
Gastroscopic pathology showed positive AFP immunohistochemistry.

The patient was a 72-year-old man who was found to have elevated blood AFP levels (1781.71ng/mL,normal range: <7.00 ng/mL) during physical examination in August 2023. No abnormalities were shown in lung CT as well as upper abdomen and urinary system color Doppler ultrasound. Five days later, the patient went to our outpatient department for treatment. The medical history was inquired, and there was no abdominal pain and diarrhea, no nausea and vomiting, no black stool, and no weight loss were reported. In addition, no previous viral hepatitis and alcoholic history was recorded. Further enhanced CT of the whole abdomen showed no tumor. The patient was instructed to have a 2-4-week follow-up visit, but he did not follow the suggestion. In November 2023, the patient returned to the hospital again because of chest tightness after physical activity. The physical examination results were as follows: T 37.4°C, P 106 times/min, R 20 times/min, BP 130/69mmHg, soft spirit, anemia, no yellow staining and bleeding points on the skin and sclera, flat and soft abdomen, no tenderness or rebound pain in the whole abdomen, and no edema in both lower limbs. After admission, further examinations were conducted (See **Table 1**), and the results showed that the blood AFP level increased to 19138.88 ng/mL. Enhanced CT of the whole-abdomen showed gastric cancer with peri-gastric and retroperitoneal lymph node metastasis. Gastroscopy pathology: poorly differentiated adenocarcinoma(stomach body)(See **Figure 1**) and immunohistochemical AFP (+) (See **Figure 2**). PET-CT: gastric cancer with peri-gastric and retroperitoneal lymph node metastasis; multiple lymph nodes were shown in the right supraclavicular area and right axilla, indicating possible metastasis; multiple low-density shadows in the right lobe of the liver, indicating possible metastasis. The final diagnosis was as follows: AFPGC with multiple metastases, gastrointestinal bleeding, and severe anemia.

**Table 1:** Admission test results.

Test	results	Reference range
leukocyte,10^9^/L	3.7	3.5-9.5
erythrocyte,10^12^/L	2.43	4.3-5.8
hemoglobin,g/L	61.0	130.0-175.0
platelet,10^9^/L	430.0	125.0-350.0
Ferritin,ng/mL	7.20	23.90-336.20
AFP,ng/mL	19138.88	<8.78
Ca724,U/mL	12.78	<6.90
CA199,U/mL	127.21	<37.00
CEA,ng/mL	3.23	<5.00
stool OB	positive	negative
ALT,U/L	15.1	9.0-50.0
AST,U/L	18.3	15.0-40.0
HBV surface antigen	negative	negative
Autoimmun Hepatitis set	negative	negative
A,C,D,E liver antibodies	negative	negative

Treatment: The patient refused surgery because of his old age and multiple metastases in the advanced stage of the tumor. He was treated with sintilimab injection immunotherapy combined with tegafur, gimeracil, and oteracil potassium capsule chemotherapy for one cycle, but the treatment was stopped because of the side effect of leukopenia (1.55 * 10 ^ 9/L). The AFP level was rechecked, and it was still greater than 10,000 IU/mL. The patient could not tolerate the treatment and abandoned it.

## Discussion

AFP, a fetal serum protein,it is produced by embryonic hepatocytes, yolk sac cells, and fetal gastrointestinal cells (5). The level of AFP in adults is very low under physiological conditions. In addition, the level of AFP is slightly increased in pregnant women, and it is generally no more than 400ng/mL. Multiple diseases can lead to elevated AFP levels (See **Table 2**). The significant elevation of AFP level is common in tumor diseases such as HCC and GCTs(testicular cancer, ovarian cancer, teratoma, etc.). Moreover, the elevation of AFP level is rare in tumor diseases such as gastric cancer and pancreatic cancer (1); The level of AFP is slightly increased in patients with viral hepatitis and liver cirrhosis, reaching generally no more than 300ng/mL.

**Table 2:** Classification of diseases causing elevated AFP (1).

Classification		cases
Tumors	common diseases	hepatocellular carcinoma (HCC)
		germ cell tumours (GCTs),(testicular cancer, ovarian cancer, teratoma, etc.)
	rare diseases	gastric cancer
		Colorectal adenocarcinoma with enteroblastic differentiation
		renal cell carcinoma
		bladder cancer
Non-tumor class	common diseases	viral hepatitis
		Liver cirrhosis
	Physiological state	pregnancy

In this patient, the level of AFP was extremely elevated, and he was finally diagnosed with gastric cancer unexpectedly. Normally, the highly specific indicators of gastric cancer are CEA, CA724 and CA199. In general, AFP is generally not routinely detected. At present, AFPGC is currently considered as a repetition of embryonic development of the digestive system. During embryonic development, the stomach and liver are derived from the primitive foregut, which is directly extended to the yolk sac. The cancer cells of the above mentioned tissues and organs have a common gene phenotype. During tumor occurrence and development, some gastric cancer cells may differentiate into hepatoid. Thus, the potential production of AFP can be expressed, and its histological morphology may be similar to hepatocellular carcinoma or yolk sac tumor. AFPGC is relatively rare in clinical practice, and its diagnosis mainly depends on pathological examination by immunohistochemical AFP (+) in primary or metastatic lesions (6). At present, AFPGC is generally divided into hepatoid type, yolk sac tumor type, and fetal gastrointestinal type (7), as well as divided into gastric hepatoid adenocarcinoma and AFP-positive adenocarcinomas without hepatoid features (8). The diagnosis of gastric hepatoid adenocarcinoma is based on the presence of hepatoid differentiation area in the tissue (9). The gastroscopic pathology of this patient showed poorly differentiated adenocarcinoma of the gastric body, immunohistochemical AFP (+), and no hepatoid differentiation.The AFP level of this patient was extremely high, and other diseases that may increase the AFP level were excluded, so the patient was diagnosed with AFPGC, belonging to AFP-positive adenocarcinomas without hepatoid features.

Compared with non-AFP-producing gastric cancer, AFPGC exhibits enhanced proliferative capacity, reduced apoptotic rate, and increased neovascularization ability (2). It is also prone to lymph node metastasis, with 33%–72% of cases developing liver metastasis (10). Most diagnoses occur at the advanced stage with a poor prognosis. At present, the treatment for AFPGC primarily revolves comprehensive therapy centered on radical gastrectomy. Apart from surgical treatment, immunotherapy, chemotherapy, and targeted therapy have been gradually used in the treatment of AFPGC, and their combined treatment can enhance the efficacy. When surgery is available, early surgical treatment can inhibit the progression of AFPGC (11); chemotherapy is the main treatment method to prolong the survival of patients with advanced AFPGC. Multi-drug combinatio chemotherapy may play a role in the treatment of AFPGC, such as tegafur, gimeracil, and oteracil potassium capsules combined with platinum or paclitaxel combined with platinum and fluorouracil. Immune checkpoint inhibitors such as programmed cell death protein 1 (PD-1) and programmed cell death ligand 1 inhibitors have shown certain efficacy in the treatment of AFPGC (12, 13). Vascular endothelial growth factor receptor is a target in AFPGC-targeted therapy research, and the targeted drugs primarily include apatinib and ramucirumab (14, 15). Some patients can be alleviated completely and persistently by using the combination of a PD-1 inhibitor (tislelizumab) and an anti-angiogenic drug (apatinib) with light toxic effects. In addition, the primary tumor and metastatic lesions gradually shrinking, and eventually disappearing, resulting in good quality of life, and progression-free survival longer than 24 months (16).

AFP level is related to the prognosis of patients. A study in 2014 (17) showed that the 1-year, 3-year, 5-year, and 10-year survival rates of AFPGC patients with AFP levels of 20-300 ng/mL were 46.7%, 28.9%, 17.8%, and 13.3%, respectively. By contrast, the survival rates of AFP patients with AFP levels >300ng/mL at 1-year, 3-year, and 5-year follow-up were 15.4%, 7.7%, and 0%, respectively. The elevation of AFP level after treatment often indicates tumor recurrence or metastasis (18). The patient opted for chemotherapy combined with immunotherapy, but he could not tolerate treatment and eventually abandoned it.

## Conclusion

AFPGC is rare in clinical practice, and the detection of AFP in gastric cancer screening is generally uncommon. Given the limited understanding, the early clinical diagnosis of AFPGC often poses challenges, leading to early missed diagnoses and delayed treatment initiation for patients. Thus, this case study aims to broaden clinicians’ perspectives on diagnosis and treatment approaches while reducing the rates of missed diagnoses and misdiagnoses. In enhancing the early detection of AFPGC and improving curative outcomes, serum AFP testing should be incorporated into gastric cancer screening protocols. In addition, postoperative monitoring of serum AFP levels can provide valuable insights into tumor recurrence and metastasis. However, elevating the standard of clinical comprehensive treatment remains pivotal in improving the overall prognosis for such patients.

## Data availability statement

The raw data supporting the conclusions of this article will be made available by the authors, without undue reservation.

## Ethics statement

The studies involving humans were approved by Huzhou Central Hospital Medical Ethics Committee. The studies were conducted in accordance with the local legislation and institutional requirements. The participants provided their written informed consent to participate in this study. Written informed consent was obtained from the individual(s) for the publication of any potentially identifiable images or data included in this article.

## Author contributions

YZ: Data curation, Formal analysis, Investigation, Writing – original draft, Writing – review & editing. JY: Data curation, Formal analysis, Investigation, Writing – original draft, Writing – review & editing. JL: Data curation, Writing – original draft. FZ: Data curation, Formal analysis, Methodology, Supervision, Writing – review & editing.
